# Use of social media in the marketing of agricultural products and farmers’ turnover in South-South Nigeria

**DOI:** 10.12688/f1000research.26353.1

**Published:** 2020-10-09

**Authors:** Henry Inegbedion, Emmanuel Inegbedion, Abiola Asaleye, Eseosa Obadiaru, Festus Asamu

**Affiliations:** 1 Department of Business Studies, Landmark University, Omu Aran, Kwara, +234, Nigeria; 2 Registry Department, Nigeria Broadcast Academy, Lagos, Lagos, +234, Nigeria; 3 Department of Economics, Landmark University, Omu Aran, Kwara, +234, Nigeria; 4 Department of Accounting and Finance, Landmark University, Omu Aran, Kwara, +234, Nigeria; 5 Department of Sociology, Landmark University, Omu Aran, Kwara, +234, Nigeria

**Keywords:** Agricultural marketing, efficiency in marketing, optimal sales turnover, social media marketing, demand for agricultural products

## Abstract

**Background:** The study investigated the use of social media in the marketing of agricultural products and farmers turnover in South-South Nigeria. The purpose of the study was to determine the extent to which the usage of social media in the marketing of agricultural products in Nigeria can enhance efficiency and farmers’ sales turnover.

**Methods:** It employed the survey research design and data were collected with the help of a structured questionnaire. Research data were analysed using t-test and least square method. **Results:** The results showed that the use of social media (Facebook, WhatsApp and Instagram) in the marketing of agricultural products enhances efficiency and turnover of farmers through a significant reduction in the cost of marketing agricultural products as well as increased awareness and the attendant increase in demand for agricultural produce.

**Conclusions: **The use of social media (WhatsApp and Instagram) in the marketing of agricultural products significantly influences cost reduction and hence efficiency in marketing as well as enhances turnover of farmers through increased demand for agricultural products. Thus, the adoption of social media in the marketing of agricultural products enhances the efficiency of agricultural marketing and sales turnover of farmers in south-south of Nigeria but only the use of WhatsApp and Instagram predict efficiency and sales turnover of farmers.

## Introduction

In many countries of the world, the agricultural sector is regarded as a stimulant of economic development ( Food and Agricultural Statistics, 2004). There is evidence to show consistently that it is not possible for any country to successfully attain industrialized status without first achieving significant success in agricultural performance since the green revolution (investment in food production) is crucial to the industrial revolution ( Inegbedion *et al*., 2020; as well as ( Inegbedion *et al*., 2018). The “agricultural sector performance, particularly through improved productivity, is one of the major ways of reducing poverty in developing countries” ( Nebo & Ejionueme, 2017), which is consistent with the key macroeconomic goals of any economy and two of the United Nation’s Sustainable Development Goals: “No Hunger” and “No Poverty”.

Over time, the agricultural sector has suffered neglect in Nigeria that it is largely unattractive to the current generation of youths. It is for this reason that a different type of agricultural marketing deserves to be given due attention to ensure that the earnings potential of agriculture is not undermined. Given the popularity of the use of social media across the different age grades in Nigeria, it is logical to expect that its use in the marketing of agricultural products will make a significant impact on the demand for agricultural products and thus on sales. This explains the rationale for this study.

## Objectives of the study

The main objective of this study was to investigate the impact of the adoption of social media in agricultural marketing by farmers in South-South Nigeria on major performance indicators Such as cost of marketing, demand for agricultural products and turnover of farmers.

## Literature review

### Channels of Advertisement

The traditional marketing of agricultural products consists of determining the farm products, producing in the right quality, storing to ensure that is the products are available, and transporting the products to the places where they are required. It consists mainly of non-verbal communication between the producer and the consumer. The producer (farmer) conceives a product that is required by consumers, produces it in a manner perceived to be consistent with customers` requirement, stores it, and distributes to locations perceived to have adequate demand. 

***Use of print and electronic media in advertising.*** Beyond the product-price-storage-distribution strategy, large organisations and government have been known to market their products through advertisements in print and electronic media. The essence of such advertisements is to create awareness of the availability of the products, the locations where they are available as well as the expected benefits of using those products, especially if the products can be substituted for other products.

***Adoption of social media for agricultural marketing in South-South Nigeria.*** Given the shortcomings of the print and electronic media in marketing agricultural products, the need for a more efficient strategy has been long overdue. The rapid growth of the mobile phone use around the world in the last few decades provided a viable marketing alternative for agricultural products in Nigeria and other emerging economies. Mobile phones have contributed significantly to the empowerment of people in developing countries in spreading information networking coverage in remote areas. Consequently, many rural areas are getting great benefit out of its usage in various spheres of endeavour. This has culminated in the improvement of the living standard of poor farmers in developed nations.

## Theoretical review

### Technology acceptance model (TAM)

 As one of the frequently used theory to underpin Internet usage, the major thrust of TAM is that the users’ beliefs in perceived usefulness and perceived ease of use of the Internet can be used to explain their acceptance of Internet ( Chuttur, 2009; Dwivedi *et al*., 2017). Thus, the extent to which the user believes that technology is useful for the cause and the extent to which he feels that he can easily use technology are critical to his/her willingness to use it. Recent modifications and refinement to TAM have culminated in the extension of its use to enable a better understanding of users` intention to use Internet technology.

### Theory of agricultural marketing cooperatives

This theory visualizes a model that studies how an agricultural marketing cooperative (AMC), specifically an AMC that is made up of many farmers as members, can adopt a direct selling approach. Under this arrangement, the farmers who are the members of the AMC can sell their products either to the cooperative or in a local market. The model shows that if the farmers decide to sell to the AMC, the decision will induce an anti-competitive effect on the direct selling market, whereas, the option of direct selling has the propensity to create a "healthy emulation" among farmers ( Agbo *et al*., 2013). This will then stimulate production and ultimately benefit the cooperative.

### Theoretical framework

This study adopts the TAM and the theory of agricultural cooperatives as its framework because the ease with which the farmers feel they can utilize social media to market their products and the usefulness of social media marketing to the attainment of their marketing goals are critical to the adoption of social media in the marketing of their products. Furthermore, the cooperative societies to which the farmers belong can play a critical role in the marketing of the farmers’ products.

### Empirical review

The empirical review focuses on cost of marketing agricultural products as well as sales turnover.

### Use of social media in marketing agricultural products and cost of marketing

Balkrishna & Deshmukh (2017) investigated the “role of social media in agriculture marketing and its scope.” They employed questionnaire and in-depth interviews to collect data. Results indicated that social media is very useful in agricultural marketing.

Vassiliadou *et al*., (2011) investigated “the use of social media among students of Technology Agriculture and their role in promoting agribusiness.” The results showed that social media usage enhances facilitation and flow of knowledge/information, as well as cheap advertisement of products.

### Use of social media in agricultural marketing and sales turnover

Mwangi & Wagoki (2016) surveyed leading media groups to investigate “the effect of social media on the performance of advertisement business in the mainstream media in Kenya.” They employed stratified random sampling to select 82 respondents. A questionnaire served as the research instrument. Descriptive and inferential statistics were employed. The findings indicate that the interactivity of social media had a significant positive relationship with the performance of the advertisement. Lashgarara *et al*., (2011) examined “ICT capabilities in improving the marketing of agricultural productions of Garmsar Township, Iran.” The survey research design was employed with questionnaire serving as the research instrument. The results indicated that ICT capabilities determined 57% variance of agricultural products marketing. Therefore, the following null hypotheses were formulated:

*H*0 _1_:  There is no significant relationship between the use of social media in agricultural marketing and cost of marketing agricultural products

*H*0 _2_ :    There is no significant relationship between the use of social media (Facebook, WhatsApp and Instagram) in agricultural marketing and farmers’ turnover from agricultural products

Other related studies are “use of information communication technologies among agricultural extension officers in Lesotho” ( Akintunde & Oladele, 2019), “adoption of agricultural e-marketing,” ( Alavion *et al*., 2017), as well as the studies of Eze *et al*. (2019), among others.

***Gap in the literature.*** Adoption of social media and other communication technologies in the marketing of agricultural products is not deficient in the empirical literature. White *et al.* (2014); Balkrishna & Deshmukh (2017) as well as Khou & Suresh (2018), investigated use of social media for agricultural marketing; Akintunde & Oladele (2019) and Alavion *et al.* (2017) examined determinants of ICT usage in the marketing of agricultural products. Jose & Lokeswari (2018) investigated users and non-users of mobile communication technologies in agricultural marketing; Lashgarara *et al.* (2011) examined ICT capabilities in improving the marketing of agricultural productions, Ogunniyi & Ojebuyi (2012) investigated “Mobile phone use for agribusiness by farmers in Southwest Nigeria while Mwangi & Wagoki (2016) investigated the effect of social media on the performance of advertisement business in in Kenya. Although a few of the studies investigated the use of social media, none appears to have focused on the implications of the usage of social media in agricultural marketing on marketing efficiency; neither is there adequate empirical evidence on how the use of social media in the marketing of agricultural products can impact on efficiency and sales turnover. Besides, related studies on the research problem in South-South Nigeria are either scant or non-existent. This study sought to fill these gaps.

## Methods

The study employed the quantitative research method. Specifically, the conclusive research design consistent with Inegbedion (2018); Inegbedion *et al*., (2018) and Inegbedion *et al*., (2016) was employed while the survey method was used in data collection. The study was conducted over July-September, 2019

### Participants

The population of the study consisted of 4280 farmers registered in cooperative societies. Of this number, 1620 are from Edo State, 1460 are from Ondo State and 1200 are from Delta state. Yamane’s formula was used to estimate a sample size of 366 ( Yamane, 1967) and proportional allocation was used to assign 139, 125 and 102 to Edo, Ondo and Delta States, respectively. Of the 366 respondents that were sampled, 246, representing 67.2% of them voluntarily participated in the study. The participants were randomly selected from farmers’ cooperative societies in the three states (a lottery method was used to achieve randomisation after stratifying the farmers on the basis of crop, poultry and fish farming). Subsequently, the farmers were contacted through any of the social media that they use (Facebook, WhatsApp or Instagram). The major inclusion criterion was membership of farmers’ cooperative while the exclusion criterion was the non-usage of any of the three social media platforms (Facebook, WhatsApp or Instagram). The choice of these states was informed mainly by convenience. Specifically, samples were taken from the current members of the cooperative societies. The sample consisted of the crop, poultry and fish farmers with evidence of usage of social media (Facebook, WhatsApp or Instagram). The sampling frame was requested from the management of the cooperative societies.

### Request for consent of respondents to participate in the study

After the sampling of the respondents, the verbal consent of the management of the cooperative societies was sought. Having obtained the consent of the leaders of the cooperatives, the consents of all the 366 sampled respondents across the three States were sought through the social media. Following the request to participate in the study, through social media 120 of them across the three states declined to participate in the study through their response to the social media message requesting their participation. While a few cited personal reasons for declining, majority of them did not advance any reason, they just refused to respond to the request. Of those approached, 246 gave their consent and thus participated in the study.

### Materials

Based on the sampling frame, a sample of respondents was selected. Thereafter, the sampled respondents were requested to participate in the study through social media. A survey was constructed and used to examine the use of social media in agricultural marketing and its implication for efficiency and sales turnover through the administration of a questionnaire, which served as the research instrument. The questionnaire contained bio-data questions and 5-point Likert scale questions dealing with social media usage in agricultural marketing and its implication for efficiency and sales turnover in South-South Nigeria. Information was elicited from the respondents via structured questionnaires through the social media channels (Facebook, Instagram and WhatsApp). The questionnaire is available as *Extended data* ( Inegbedion *et al*., 2020).

### Validity of instrument

A pilot test was conducted on 20 of the sampled respondents. Based on the results obtained from the pilot test, validity and reliability of the instrument were determined. For validity, two approaches were used. First, the instrument was given to experts in management and marketing in the authors’ institution for their expert opinion, this served to fulfil the condition for face validity. Thereafter, content validity index (CVI) was computed. Both scale and item content validity measures were used. The results obtained were 0.66 for scale and 0.67 and 0.67 item CVI of use of social media in marketing agricultural products and cost reduction as well as use of social media in the marketing of agricultural products and farmers’ turnover respectively, thus showing that the instrument was valid since all the values are approximately 0.7 and a value of 0.7 is indicative of a valid instrument (see Table 1).

**Table 1.** Content validity index.

Construct	Item-CVI	Scale-CVI
Use of social media and cost reduction	0.67	
Use of social media and sales turnover	0.66	
Entire Instrument		0.67

### Reliability of the instrument

Cronbach’s alpha (α) was used to determine the reliability of the instrument. The computed Cronbach alpha values were 0.70 and 0.75 for use of social medial and cost reduction as well as use of social media and sales turnover respectively while the computed alpha of the entire instrument was 0.84. These values were considered significant since they are approximately 0.7 or more, thus indicating that the items in the instrument are internally consistent. In other words, the instrument is reliable. The foregoing indicates that the instrument is valid and reliable (see Table 2).

**Table 2.** Instrument reliability.

Construct	Cronbach alpha of Constructs	Cronbach alpha of the entire instrument
Use of social media and cost reduction	0.70	
Use of social media and sales turnover	0.75	
Entire Instrument		0.84

### Model specification

CMAP  =  f (UFB, UWA and UINS)            . . .             (1)

DAP    =  f (UFB, UWA and UINS)            . . .              (2)

In specific terms, equations 1 and 2 yield

CMAP  = *β*_0_ + *β*_1_ UFB + *β*_2_ UWA + UINS+ e      . . .     (3)

DAP    = *β*_0_ + *β*_1_ UFB + *β*_2_ UWA + UINS + e      . . ..    (4)

Where

      CMAP = Cost of marketing agricultural products;

      DAP = Demand for agricultural products;

      UFB = usage of Facebook;

      UWA = usage of WhatsApp;

      UINS = usage of Instagram; and

      e. = random error observed along with the variables

### Statistical analysis

Research data were analysed using the one-sample t-test and least-squares regression. One sample t-test was used to test for significance of the usage of social media constructs in cost reduction and sales turnover while the least-squares method was used to test for the predictive power of the entire constructs (collectively) with respect to cost reduction and sales turnover; besides, the signs of the coefficients of the constructs in the regression model were used to infer the direction of the relationships between usage of social media in agricultural marketing and cost of marketing agricultural products on one hand as well as usage of social media in agricultural marketing and sales turnover on the other hand. Data analysis was conducted with SPSS 20.

## Ethical approval

The authors sought and got ethical approval to conduct the study from the Landmark University Research Ethical Board. Furthermore, the authors complied with conventional ethical standards in conducting the study, including the request for the consent of the sampled respondents to participate in the study.

## Results

The results focus on the impact of social media usage on cost reduction in the marketing of agricultural products and on sales turnover of the farmers. *Underlying data* are available from Dryad ( Inegbedion *et al*., 2020).

### Social media usage and reduction in cost of advertising agricultural products

A comparison of the usage of Facebook in agricultural marketing with cost reduction in marketing of agricultural products revealed that respondents who agreed that the usage of Facebook enhances cost reduction had a mean score 3.11. Given the five-point Likert scale, a test value of 3 was used. The computed t- and p-values were 2.04 and 0.046 respectively. This shows that the test was significant at P<0.05. We thus conclude, at the 95% confidence level, that usage of Facebook for marketing agricultural products significantly reduces the cost of marketing (see Table 3).

**Table 3.** Use of social media in marketing agricultural products and cost reduction.

Channel	Mean	SD	Mean Diff	Test Value	T	Sig. 2-tailed	N
Facebook	3.11	0.41	0.11	3.00	2.04	0.046	246
WhatsApp	3.59	0.63	0.59	3.00	14.58	0.000	246
Instagram	3.09	0.454	0.09	3.00	1.99	0.048	246

A comparison of the usage of WhatsApp in agricultural marketing with cost reduction in marketing of agricultural products revealed that respondents who agreed that the usage of Facebook enhances cost reduction had a mean score 3.59. Given the five-point Likert scale, a test value of 3 was used. The computed t- and p-values were 14.58 and 0.001, respectively. This shows that the test was significant at the one per cent level. We thus conclude, at the 99% confidence level, that usage of WhatsApp for marketing agricultural products significantly reduces the cost of marketing (see Table 3).

A comparison of the usage of Instagram in agricultural marketing with cost reduction in marketing of agricultural products revealed that respondents who agreed that the usage of Facebook enhances cost reduction had a mean score 3.09. Given the five-point Likert scale, a test value of 3 was used. The computed t and p values were 1.99 and 0.048, respectively. This shows that the test was significant at the five per cent level. We may thus conclude, at the 95% confidence level, that usage of Instagram for marketing agricultural products significantly reduces the cost of marketing (see Table 3).

A regression model of usage of social media in the marketing of agricultural products and cost of marketing agricultural products revealed that the R ^2^ value was 0.48, thus implying that 48% of the variation in the cost of marketing agricultural products is explained by variation in the usage of social media channels (see Table 4). The ANOVA table shows a calculated F of 10.94 with an associated significant probability of p < 0.001, thus indicating that the F value is significant. The implication is that the overall significance of the model is good (see Table 4). The regression coefficients show that the computed t-values and associated p-values were 3.152 (0.002), 0.337 (0.076), 5.015 (p < 0.001) and 2.570 (0.011) for constant, Facebook usage, WhatsApp usage and Instagram usage, respectively (see Table 4). The implication is that the explanatory variables, consisting of the usage of social media channels (WhatsApp and Instagram) are significant and are thus predictors of cost reduction in the marketing of agricultural products. However, Facebook usage is not significant and is thus not a predictor of cost reduction in the marketing of agricultural products. 

**Table 4.** Usage of social media and cost of marketing agricultural products.

Model Summary						
R	R-square	Adjusted R Square	Std. Error of Estimate	Durbin-Watson		
0.694	0.48	0.43	0.707	1.67		
**ANOVA**						
F =10.935			Sig. = 0.000			
**Coefficients**						
**Model**		**B**	**Std Error**	**Beta**	**t**	**Sig**
Constant		1.334	0.423		3.152	0.002
Facebook cost reduction		0.020	0.059	0.020	0.317	0.076
WhatsApp cost reduction		0.360	0.071	0.304	5.015	0.000
Instagram cost reduction		0.257	0.100	0.155	2.570	0.011

### Social media usage and sales turnover from agricultural products

A comparison of the usage of Facebook in agricultural marketing with sales turnover of farmers revealed that respondents who agreed that the usage of Facebook enhances farmers’ sales turnover had a mean score 3.214. Given the five-point Likert scale, a test value of 3 was used. The computed t and p values were 5.44 and P <0.001 respectively. This shows that the test was significant at p < 0.05. We thus conclude, at the 95% confidence level, that usage of Facebook for marketing agricultural products significantly enhances the sales turnover of farmers (see Table 5). 

**Table 5.** Use of social media in marketing agricultural products and sales turnover.

Channel	Mean	SD	Mean Diff.	Test Value	t	Sig. 2-tailed	N
Facebook	3.214	0.62	0.214	3.00	5.44	0.000	246
WhatsApp	3.289	0.78	0.289	3.00	5.84	0.000	246
Instagram	3.115	0.418	0.115	3.00	4.32	0.000	246

A comparison of the usage of WhatsApp in agricultural marketing with sales turnover of agricultural products revealed that respondents who agreed that the usage of Facebook enhances sales turnover had a mean score 3.289. Given the five-point Likert scale, a test value of 3 was used. The computed t and p values were 5.84 and 0.000, respectively. This shows that the test was significant at the one per cent level. We thus conclude, at the 99% confidence level, that usage of WhatsApp for marketing agricultural products significantly optimises the sales turnover of farmers (see Table 5).

A comparison of the usage of Instagram in agricultural marketing with sales turnover of agricultural products revealed that respondents who agreed that the usage of Facebook enhances sales turnover had a mean score 3.115. Given the five-point Likert scale, a test value of 3 was used. The computed t and p values were 4.32 and P < 0.001 respectively. This shows that the test was significant at P < 0.01. We thus conclude, at the 99% confidence level, that usage of Instagram in marketing agricultural products significantly optimises the sales turnover of farmers (see Table 5).

A regression model of usage of social media and turnover of agricultural products revealed that the R ^2^ value was 0.58, thus implying that 58% of the variation in turnover of agricultural products is explained by variation in the usage of social media (see Table 6). The ANOVA table shows a calculated F of 7.21 with an associated significant probability of P < 0.001. The implication is that the overall significance of the model is good (see Table 6). Lastly, the regression coefficients show that the computed t and associated significant probabilities were 12.58 (0.00), 0.816 (0.415), 2.77 (0.006) and 4.614 (p < 0.001) for constant, Facebook usage, WhatsApp usage and Instagram usage, respectively (see Tables 6). The implication is that WhatsApp and Instagram usage are predictors of turnover of agricultural products. 

**Table 6.** Usage of social media and turnover of agricultural products.

Model Summary								
R	R-square	Adjusted R Square	Std. Error of Estimate	Durbin-Watson			
0.76	0.58	0.47	0.528	1.966			
**ANOVA**							
F =7.209			Sig. = 0.000				
**Coefficients**								
Model			B	Std Error	Beta	t	P-value
Constant			2.436	0.264		18.79	0.000
Facebook Turnover			0.038	0.044	0.82	0.816	0.415
WhatsApp Turnover			0.123	0.044	0.254	2.77	0.006
Instagram Turnover			0.322	0.070	0.358	4.614	0.000

### Demographic variables and social media usage

F test was conducted to compare respondents’ perception of the use of social media in the marketing of agricultural products with their demographic variables. A comparison of respondents’ perception of usage of social media in cost reduction and demographic variables had the following computed F and associated significant probabilities 1.12 (0.348), 1.36 (0.25), 0.673 (0.671) and 1.28 (0.28) for age, sex, educational qualification and farm categories, respectively. The implication is that respondents’ perception of the significance of the usage of social media in the reduction of agricultural marketing cost is not influenced by demographic variables (see Table 7).

**Table 7.** Demographic variables and respondents’ perception of cost reduction.

	Age	Sex	Educational Qualification	Farm Category
F	1.12	1.36	0.673	1.28
Sig.	0.348	0.25	0.671	0.28

Lastly, a comparison of respondents’ perception of the significance of the usage of social media in enhancing the turnover of agricultural products had the following computed F and associated p-values 0.29 (0.83) 1.26 (0.27), 0.961 (0.51) and 0.734 (0.73) for age, sex, educational qualification and farm categories, respectively. The implication is that respondents’ perception of the significance of the usage of social media in enhancing the turnover of agricultural products is not influenced by demographic variables (see Table 8).

**Table 8.** Demographic variables and respondents’ perception of turnover.

	Age	Sex	Educational Qualification	Farm Category
F	0.29	1.26	0.961	0.734
Sig.	0.83	0.27	0.51	0.730

## Discussion

Results of the study indicate that the use of Facebook, WhatsApp and Instagram in the marketing of agricultural products has a significant influence on the reduction of the cost of marketing agricultural products. However, while the use of WhatsApp and Instagram were found to be significant predictors of reduction in the cost of marketing agricultural products, the use of Facebook was not found to be significant. The implication of the significance of social media channels (WhatsApp and Instagram) in the reduction of the cost of marketing agricultural products is that the use of social media channels in the marketing of agricultural products enhances efficiency in the marketing of agricultural products. The results are consistent with the findings of Balkrishna & Deshmukh (2017); Mwangi & Wagoki (2016); Ogunniyi & Ojebuyi (2012); White *et al*., (2014); as well as Vassiliadou *et al*., (2011). Results of the study further indicate that the use of social media channels (Facebook, WhatsApp and Instagram) in the marketing of agricultural products has a significant influence on the enhancement of the turnover of farmers. However, just like in the previous model, the use of Facebook did not demonstrate a significant predictive power on sales turnover. Thus, only the use of WhatsApp and Instagram have strong predictive powers on sales turnover. The results are consistent with the findings of Mwangi and Wagoki, Khou & Suresh (2018) as well as Ogunniyi & Ojebuyi (2012).

The first regression model shows that WhatsApp and Instagram usage in the marketing of agricultural products are significant predictors of cost reduction in the marketing of agricultural products while Facebook was not significant. Thus, usage of WhatsApp and Instagram in the marketing of agricultural products has significant implications on the marketing of agricultural products. This tends to suggest that the usage of Facebook by farmers in the marketing of agricultural products is minimal compared to the usage of WhatsApp and Instagram. The coefficient of determination of 0.48 shows that 48% of the variation in advertising cost of farmers is attributable to the usage of social media in advertising. In the same vein, the second regression model shows that the usage of WhatsApp and Instagram are significant predictors of enhanced sales turnover from agricultural products because the two channels of social media help in speedy dissemination of information about the agricultural products to a wide audience of customers, thus leading to increased demand on the short-run. The two regression models indicate that WhatsApp and Instagram are predictors of efficiency of cost of marketing agricultural products and sales turnover from agricultural products.

### Proposed model of usage of social media and expected outcomes

**Figure 1.**
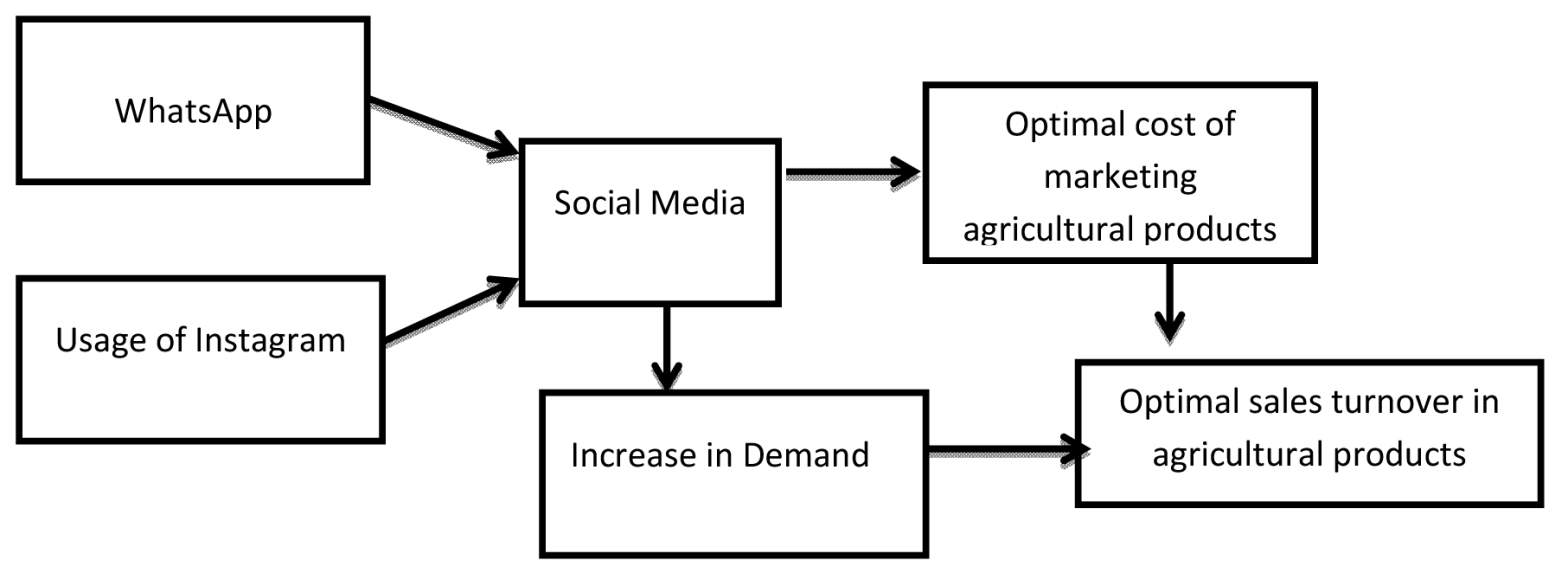
A model of social media marketing of agricultural products and sales turnover.

Based on the research findings, a model of social media marketing of agricultural products, efficiency of agricultural marketing and sales turnover was proposed. The model shows that two social media channels (WhatsApp and Instagram) predict social media impact on the marketing of agricultural products in terms of efficiency and enhanced turnover. The model shows that the use of social media (WhatsApp and Instagram) leads to optimal marketing cost and thus efficient marketing of agricultural products. The optimal cost of marketing agricultural products translates to cost savings and hence optimal turnover in agricultural products. Also, effective use of social media (WhatsApp and Instagram) stimulates demand which also results in optimal sales turnover from agricultural products (see Figure 1).

## Conclusions

Given the problem definition and findings, the research concludes that: The use of social media (Facebook, WhatsApp and Instagram) in the marketing of agricultural products significantly influences cost reduction and hence efficiency in marketing as well as enhances turnover of farmers through increased demand for agricultural products. Thus, the adoption of social media in the marketing of agricultural products enhances the efficiency of agricultural marketing and sales turnover of farmers in south-south of Nigeria but only the use of WhatsApp and Instagram predicts efficiency of agricultural marketing and sales turnover of farmers.

This study has made significant contributions to marketing and management knowledge. First, it is among the few studies that have examined the influence of social media marketing on marketing efficiency and farmers’ sales turnover in Nigeria. Secondly, it is among the very few studies to have studied the adoption of social media in agricultural marketing in south-south Nigeria. Thus, a major point of departure of this study from previous studies is its examination of the implications of the usage of social media usage in agricultural marketing on marketing efficiency and sales turnover. It has thus made a concerted effort in reinforcing the interest of farmers in the use of social media in the marketing of agricultural products in south-south Nigeria.

The study is not without limitations which indicate the need for further studies to minimize the constraints associated with this study. Out of six South-South states in Nigeria, only three were studied. It is uncertain if the three states will adequately represent the six states in the region. The tendency of the three states to inadequately represent the six South-South states is a limitation to the results of this study. Furthermore, the use of only members of the farmers’ cooperative society constitutes a limitation to the results of the study. Farmers who do not belong to the cooperative society may have some dissenting perceptions from those of the members of a cooperative society.

### Implication of findings/recommendation

The significance of the use of social media in the marketing of agricultural products to marketing efficiency and sales turnover implies that policy makers in government and strategic managers of agro-allied firms can embrace the use of social media in the marketing of agricultural products to minimize the cost of marketing and enhance turnover from the sales of agricultural products. Growth in the agricultural sector has implications on a nation’s gross domestic product and by implication, on national development. Given the problem definition and research findings, the following recommendations are suggested. Policymakers in government should be concerned about increasing agricultural production as well as the marketing of agricultural products to enhance earnings from agricultural production. This will attract many unemployed youths to the agricultural sector and thus help to guarantee food security as well as drastically reduce the level of unemployment in the country. Consequently, policymakers in government and other stakeholders like managers of cooperative societies and other farmers’ associations should promote the adoption of social media in agricultural marketing through sensitization of the farmers as well as the outright provision of modern communication gadgets to farmers at subsidized prices.

Future studies should attempt to minimize limitation associated with cultural bias by including more south-south states in the sample as well as try to include non-members of the farmers’ cooperative society in the sample.

## Data availability

### Underlying data

Dryad: Use of social media in the marketing of agricultural products and farmers’ turnover in South-South Nigeria. https://doi.org/10.5061/dryad.jwstqjq76 ( Inegbedion *et al*., 2020).

File ‘Data-Use_of_Social_media_in_Agric_Markt-22’ contains basic demographic information, questionnaire responses and social media use data from each participant.

### Extended data

Dryad: Use of social media in the marketing of agricultural products and farmers’ turnover in South-South Nigeria. https://doi.org/10.5061/dryad.jwstqjq76 ( Inegbedion *et al*., 2020).

File ‘Questionnaire-Use_of_Social_Media_in_Agric_Marketing.docx’ contains a blank copy of the questionnaire used in this study.

Data are available under the terms of the Creative Commons Zero "No rights reserved" data waiver (CC0 1.0 Public domain dedication).

## References

[ref-1] Agbo M Rousselière D Salanié J: A Theory of Agricultural Marketing Cooperatives with Direct Selling. Working Papers 1331, Groupe d'Analyse et de Théorie Economique Lyon St-Étienne (GATE Lyon St-Étienne): Université de Lyon.Handle: *RePEc:gat:wpaper: 1331*.2013.

[ref-2] Akintunde MAO Oladele OI: Use of Information Communication Technologies among Agricultural Extension Officers in Lesotho. *Journal of Agricultural Extension.* 2019;23(3):50–65. 10.4314/jae.v23i3.4

[ref-3] Alavion SJ Allahyari MS Al-Rimawi AS: Adoption of agricultural e-marketing: Application of the theory of planned behaviour. *Journal of International Food Agribusiness Marketing.* 2017;29(1):1–15. 10.1080/08974438.2016.1229242

[ref-5] Balkrishna BB Deshmukh AA: A study on the role of social media in agriculture marketing and its scope. *Global Journal of Management and Business Research: E-Marketing.* 2017;17(1):1–5. Reference Source

[ref-30] Chuttur M: Overview of the technology acceptance model: Origins, developments and future direction.All Sprouts Content.2009;290. Reference Source

[ref-31] Dwivedi YK Rana N Jeyaraj A: Re-examining the unified theory of acceptance and use of technology (UTAUT): Towards a revised theoretical model. *Information Systems Frontiers.* 2017. 10.1007/s10796-017-9774-y

[ref-7] Eze SC Chinedu-Eze VC Bello A: Mobile marketing technology adoption in service SMEs: a multi-perspective framework. *Journal of Science and Technology Policy Management.* 2019;10(3):569–596. 10.1108/JSTPM-11-2018-0105

[ref-8] FAOSTAT: Online Statistics from the Food and Agriculture Organisation of the United Nations(FAO).2004.

[ref-9] Inegbedion HE Obadiaru E Obasaju B: Financing Agriculture in Nigeria through Agricultural Extension Services of Agricultural Development Programmes (ADPs) [version 3; peer review: 2 approved]. *F1000Res.* 2018;7:1833. 10.12688/f1000research.16568.3 31231505 PMC6556997

[ref-10] Inegbedion HE: Factors that Influence Customers’ Attitude toward Electronic Banking in Nigeria. *Journal of Internet Commerce.* 2018;17(4):325–338. 10.1080/15332861.2018.1463482

[ref-11] Inegbedion HE Obadiaru DE Bello DV: Factors that influence consumers’ attitude towards internet buying in Nigeria. *Journal of Internet Commerce.* 2016;15(4):353–375. 10.1080/15332861.2016.1252646

[ref-12] Inegbedion H Inegbedion E Asaleye A: Use of social media in the marketing of agricultural products and farmers’ turnover in South-South Nigeria, v2, Dryad, Dataset.2020. 10.5061/dryad.jwstqjq76 PMC8356261 34394922

[ref-13] Jose AM Lokeswari K: A study on users and non-users of ICT among the farming community. *Global Media Journal.* 2018;16(31):1–5. Reference Source

[ref-14] Khou A Suresh KR: A Study on the role of social media mobile applications and its impact on agricultural marketing in Puducherry Region. *J Manage.* 2018;5(6):28–35. 10.18782/2320-7051.7722

[ref-15] Lashgarara F Mohammadi R Najafabadi MO: ICT capabilities in improving the marketing of agricultural productions of Garmsar Township, Iran. *Annals of Biological Research.* 2011;2(6):356–363. Reference Source

[ref-17] Nebo GN Ejionueme N: Adopting Agricultural Marketing Approach for Improving Agricultural Sector Performance in Nigeria. *Journal of Business and Management.* 2017;19(4):4–17. Reference Source

[ref-18] Ogunniyi MD Ojebuyi BR: Mobile Phone Use for Agribusiness by Farmers in Southwest Nigeria. *Electronic Journals Service(EJS).* 2012;20(10):172–187. Reference Source

[ref-21] Mwangi MW Wagoki J: Effect of social media on the performance of advertisement business in the mainstream media in Kenya: A survey of leading media groups in Kenya. *International Journal of Economics, Commerce and Management.* 2016;4(4):159–177. Reference Source

[ref-22] Vassiliadou S Vogiatzi M Amygdalas T: The use of social media among students of Technology Agriculture and their role in promoting agribusiness. In: M. Salampasis, A. Matopoulos(eds.): *Proceedings*of the International Conference on Information and Communication Technologies for Sustainable Agri-production and Environment(HAICTA 2011): Skiathos.2011. Reference Source

[ref-23] White D Meyers C Doerfert D: Exploring agriculturalists' use of social media for agricultural marketing. *Journal of Applied Communications.* 2014;98(4):1–14. Reference Source

[ref-24] Yamane T: Statistics, an introductory analysis, Harper and Row.1967. Reference Source

