# Supplementary material for: Use of social media in the marketing of agricultural products and farmers’ turnover in South-South Nigeria
Source: F1000Res. 2021 Mar 12;9:1220. Originally published 2020 Oct 9. [Version 2] doi: 10.12688/f1000research.26353.2 (PMC8356261; doi:10.12688/f1000research.26353.2)
Supplement: Peer review history

# Peer review: Reviewer response for version 2

**DOI:** 10.5256/f1000research.54968.r86340

**Published:** 2021-08-10

**Authors:** K. Lokeswari

**Affiliations:** 1 Bharathiar University, Coimbatore, Tamil Nadu, India

This research study on the use of social media in the marketing of agricultural products and farmers turnover in South-South Nigeria followed the research procedures perfectly. Reviews are arranged in topic order. In-depth analysis has been done to retrieve the result. In conclusion information on social media would have given more.

 Overall the report looked crisp and neat. The figure title should appear at the bottom of the figure and the table title should appear at the top of the table.

 The page number is superfluous.

 Overall this is a well written and well organized report. The content of the paper is well organized and concise.

 All relevant information is given where ever it is necessary.

 So strongly I recommend this article for publishing in your journal.

Is the work clearly and accurately presented and does it cite the current literature?

Yes

If applicable, is the statistical analysis and its interpretation appropriate?

Yes

Are all the source data underlying the results available to ensure full reproducibility?

Yes

Is the study design appropriate and is the work technically sound?

Yes

Are the conclusions drawn adequately supported by the results?

Partly

Are sufficient details of methods and analysis provided to allow replication by others?

Yes

Reviewer Expertise:

My area of research is Development Communication & Information Communication Technology.

I confirm that I have read this submission and believe that I have an appropriate level of expertise to confirm that it is of an acceptable scientific standard.

# Peer review: Reviewer response for version 2

**DOI:** 10.5256/f1000research.54968.r81369

**Published:** 2021-03-19

**Authors:** Phillip Alege

**Affiliations:** 1 Department of Economics and Development Studies, Covenant University, Ota, Nigeria

1. Under Literature Review, 2.2.3 'Theoretical Framework' should move to the beginning of Section 3 as a preamble.
2. 3.1 should be Study Population and not the paragraph.
3. The Sub-sections under Section 4 should be numbered properly.
4. Number "Discussion" as 5 and "Conclusion" as 6.
5. Everything about "Data Availability" should go back to Sub-section 3.2 Data Sources.

With this, the paper is good to go.

Is the work clearly and accurately presented and does it cite the current literature?

Yes

If applicable, is the statistical analysis and its interpretation appropriate?

Yes

Are all the source data underlying the results available to ensure full reproducibility?

Yes

Is the study design appropriate and is the work technically sound?

Yes

Are the conclusions drawn adequately supported by the results?

Yes

Are sufficient details of methods and analysis provided to allow replication by others?

Yes

Reviewer Expertise:

NA

I confirm that I have read this submission and believe that I have an appropriate level of expertise to confirm that it is of an acceptable scientific standard.

# Peer review: Reviewer response for version 1

**DOI:** 10.5256/f1000research.29091.r73490

**Published:** 2021-02-23

**Authors:** Phillip Alege

**Affiliations:** 1 Department of Economics and Development Studies, Covenant University, Ota, Nigeria

**Abstract:**

 The Abstract is too long. It should be brief and sharp. The content should include the following: background/motivation; objective of the paper, methodology, data, results and interpretation. It must also include contribution to knowledge. This component of the study should not be more than 250 words.

**Introduction:**

 This is well written. However, the authors should take another look at line three and check the sentence for consistency. It should be “there are evidences….” You cannot use only one observation to conclude on an issue. Also, there is no need to create a sub-section for “Objective of the Study”

**MAJOR COMMENTS**

 This is mainly on the Structure of the paper. I will suggest the Sections should be numbered for ease of referencing between the different parts of the report. This is also important for the readers.

 An insight into this is given in what follows:

1. Literature Review
  1. Channels of advertisement
  2. Theoretical Review
    1. Technology acceptance model (TAM)
    2. Theory of agricultural marketing cooperatives
  3. Empirical review
    1. Use of social media in marketing agricultural products and cost of marketing
    2.    Use of social media in agricultural marketing and sales turnover

      

1. Methods
  1. Study Population: Issues of request for consent, validity and Reliability must be discussed here.
  2. Model Specification
  3. Estimation Technique instead of Statistical Analysis
  4. Data Sources instead of Materials and Ethical Matters should be here
2. Estimation Results and Discussion
  1. Results
  2. Discussion
3. Conclusion

**MINOR**

 There are a few typographical errors to be attended to. The authors should check the soft copy.

**GENERAL COMMENTS**

 The paper addresses a very pertinent question in this era of New Normal on the need for the adoption of social media in the marketing of agricultural products. The recommendations are the ways to go. However, if the idea captured in **“Figure 1: A model of social media marketing of agricultural products and sales turnover” **is a result emanating from this study, then it should be discussed earlier under Section 4.2.

 Besides, the paper is well written and had made a tangible contribution to knowledge.

Is the work clearly and accurately presented and does it cite the current literature?

Yes

If applicable, is the statistical analysis and its interpretation appropriate?

Yes

Are all the source data underlying the results available to ensure full reproducibility?

Yes

Is the study design appropriate and is the work technically sound?

Yes

Are the conclusions drawn adequately supported by the results?

Yes

Are sufficient details of methods and analysis provided to allow replication by others?

Yes

Reviewer Expertise:

NA

I confirm that I have read this submission and believe that I have an appropriate level of expertise to confirm that it is of an acceptable scientific standard, however I have significant reservations, as outlined above.

# Response

**Published:** 2021-02-24

**Authors:** Henry INEGBEDION

**Affiliations:** Landmark University, Nigeria

**Comment:**       The Abstract is too long. It should be brief and sharp. The content should include the following: background/motivation; objective of the paper, methodology, data, results and interpretation. It must also include contribution to knowledge. This component of the study should not be more than 250 words.

**Response**:        F1000Research provided the template for the structure of abstract. In any case, the abstract has been reduced to below 250 words as suggested

**Comment**:       This is well written. However, the authors should take another look at line three and check the sentence for consistency. It should be “there are evidences….” You cannot use only one observation to conclude on an issue. Also, there is no need to create a sub-section for “Objective of the Study”

**Response**:        The sentence has been revised for consistency and the section on “objective of the study” has been removed as suggested

**Comment**:       This is mainly on the Structure of the paper. I will suggest the Sections should be numbered for ease of referencing between the different parts of the report. This is also important for the readers. An insight into this is given in what follows **:**

**Response**:        The paper has been restructured and the sections of the paper have been numbered as suggested by the reviewer 

**Comment**: **There are a few typographical errors to be attended to. The authors should check the soft copy.**

**Response**:        These have been worked on

**Comment**: **A model of social media marketing of agricultural products and sales turnover” is a result emanating from this study, then it should be discussed earlier under Section 4.2** **.**

 Response:        This section has been merged with section 4.2 as suggested

# Peer review: Reviewer response for version 1

**DOI:** 10.5256/f1000research.29091.r76594

**Published:** 2021-02-16

**Authors:** Lauri M Baker

**Affiliations:** 1 Agricultural Communication, University of Florida, Gainesville, Florida, 32611, USA

This study is extremely interesting with regard to understanding social media use in agriculture in Nigeria and contributes to the body of knowledge more largely in the area of marketing agricultural products online.

 Since this is the first time I am reviewing for this journal, I wasn’t sure what to expect, but there is a fair amount of copyediting that needs to be done in this manuscript. I have made note of some major issues here in my review, but there are others. Also, perhaps it is acceptable in this journal, but I was trained that in research writing each paragraph needs a minimum of three sentences, and some sentences in this article are shorter than that. Additionally, some of the language used within the article isn’t inclusive. I know many journals now have a stance on this, I am not sure if this journal does or not. The present article uses “he” or he/she instead of they or other neutral pronouns in some places.

 I have the following specific feedback:

- The end of the second sentence of the manuscript isn’t finished. It ends with “as well as”.
- The first paragraph and first sentence of the literature review has a grammatical error in this section “ensure that is the products are available”. It is unclear exactly what the authors mean because of the sentence structure.
- The first sentence under theoretical framework – theory should be theories.
- Internet is chaptalized throughout, but it is no longer considered a proper noun in most style formats.
- The first sentence of the theoretical framework is difficult to understand. I see the authors are trying to convey how they applied this framework, but how that was done is unclear as written.
- The majority of the empirical review section lacks flow and is choppy. It seems the authors have made more of an annotated bibliography instead of synthesizing and apply how each of these studies relates to their present work.
- Participants were described in present tense instead of past tense. This section was also missing commas in the numbers.
- Sampling procedure was explained well and was adequate, but again the writing in this section should be improved.
- I appreciate that the reliability is presented so clearly for each construct, but Tables 1 and 2 are overly complicated. I don’t think you need the 3 ^rd^ column if you adjust the headings accordingly.
- “analysed” is misspelled. It should be analyzed.
- There are places in the results where a capital P is used for significance level. In my training typically a lowercase p is used as in p < .05 and the authors do use this in some cases. I would advocate for the lowercase, but above all consistency.
- Rounding in the results section is not consistent.
- The use of the Oxford comma in this manuscript is not consistent.
- The conclusions and discussions and implications make sense from the results presented, and I believe the findings provide value in this context. However, the writing in these final sections is difficult to follow and has similar grammatical and flow areas, like the rest of the manuscript.

I would like to see the authors do some serious work in the writing of this manuscript. I hate to reject any article that clearly represents a solid study that can add to the literature in this area, but MAJOR improvements are needed in writing quality, editing, and flow.

Is the work clearly and accurately presented and does it cite the current literature?

Partly

If applicable, is the statistical analysis and its interpretation appropriate?

Yes

Are all the source data underlying the results available to ensure full reproducibility?

Yes

Is the study design appropriate and is the work technically sound?

Yes

Are the conclusions drawn adequately supported by the results?

Yes

Are sufficient details of methods and analysis provided to allow replication by others?

Yes

Reviewer Expertise:

Agricultural communication, social media strategy, marketing

I confirm that I have read this submission and believe that I have an appropriate level of expertise to confirm that it is of an acceptable scientific standard, however I have significant reservations, as outlined above.

# Response

**Published:** 2021-02-24

**Authors:** Henry INEGBEDION

**Affiliations:** Landmark University, Nigeria

**Comment**:       but there is a fair amount of copyediting that needs to be done in this manuscript.

**Response**:        Significant copyediting has been done

**Comment**:       Also, perhaps it is acceptable in this journal, but I was trained that in research writing each paragraph needs a minimum of three sentences, and some sentences in this article are shorter than that.

**Response**: The paragraphs have been adjusted accordingly

**Comment**:       Additionally, some of the language used within the article isn’t inclusive. I know many journals now have a stance on this, I am not sure if this journal does or not. The present article uses “he” or he/she instead of they or other neutral pronouns in some places.

**Response**:        Efforts have been made to enhance the inclusiveness of the language used

**Comment**:              I have the following specific feedback:

        The end of the second sentence of the manuscript isn’t finished. It ends with “as well as”.

**Response**:        This has been corrected

**Comment**:       The first paragraph and first sentence of the literature review has a grammatical error in this section “ensure that is the products are available”. It is unclear exactly what the authors mean because of the sentence structure

**Response**:        This has been corrected - “ensure that is the products are available”

**Comment**:      The first sentence under theoretical framework – theory should be theories.

**Response**:        This has been corrected

**Comment**:       Internet is chaptalized throughout, but it is no longer considered a proper noun

                               in most style formats.

**Response**:        This has now been corrected

**Comment**:       The first sentence of the theoretical framework is difficult to understand. I see the authors are trying to convey how they applied this framework, but how that was done is unclear as written.

 Response: It is actually an attempt to justify the use of the two theories as framework, clarification has been provided. The how has been explained in the theoretical review.

**Comment**:       The majority of the empirical review section lacks flow and is choppy. It seems the authors have made more of an annotated bibliography instead of synthesizing and apply how each of these studies relates to their present work

**Response**:        Some level of synthesis has been provided to link the empirical literature to the study

 Comment:       Participants were described in present tense instead of past tense. This section was also missing commas in the numbers.

            

**Response**:        The tenses and the missing comas have been corrected

**Comment**:       Sampling procedure was explained well and was adequate, but again the writing in this section should be improved.

**Response**:        The section has been improved upon

**Comment**:       I appreciate that the reliability is presented so clearly for each construct, but Tables 1 and 2 are overly complicated. I don’t think you need the 3rd column if you adjust the headings accordingly.

 Response:        The headings have been adjusted and the third column has been removed

**Comment**:       “analysed” is misspelled. It should be analyzed

**Response**:        This has been corrected

**Comment**:       There are places in the results where a capital P is used for significance level. In my training typically a lowercase p is used as in p < .05 and the authors do use this in some cases. I would advocate for the lowercase, but above all consistency.

**Response**:        This has been corrected

**Comment**:       Rounding in the results section is not consistent

**Response**:        Done

**Comment**:              The use of the Oxford comma in this manuscript is not consistent.

**Response**:        This has been addressed

**Comment**:       The conclusions and discussions and implications make sense from the results presented, and I believe the findings provide value in this context. However, the writing in these final sections is difficult to follow and has similar grammatical and flow areas, like the rest of the manuscript.

            

**Response**:        The grammatical flaws have been significantly addressed

**Comment**:       MAJOR improvements are needed in writing quality, editing, and flow

**Response**:        The quality of the paper has been enhanced
